# Supplementary material for: Methods for Stratification and Validation Cohorts: A Scoping Review
Source: J Pers Med. 2022 Apr 26;12(5):688. doi: 10.3390/jpm12050688 (PMC9144352; doi:10.3390/jpm12050688)
Supplement: Supplementary file 1 [file jpm-12-00688-s001.zip › jpm-1611778-supplementary.pdf]

Table S1. Search strategies applied

Table S1a. Alzheimer’s Disease Searches. Pubmed 15/5/2020

No.	Query	Results
#14	#9 OR #13 Sort by: Publication Date	56
#13	#7 AND #12 Sort by: Publication Date	45
#12	"systematic review*" Sort by: Publication Date	189,382
#9	#1 AND #2 AND #3 AND #6 Filters: Meta-Analysis, Review Sort by: Publication Date	27
#7	#1 AND #2 AND #3 AND #6 Sort by: Publication Date	1,86
#6	#4 OR #5 Sort by: Publication Date	231,788
#5	dementia[Title/Abstract] OR alzheimer[Title/Abstract] Sort by: Publication Date	213,425
#4	("Dementia"[Mesh:NoExp]) OR "Alzheimer Disease"[Mesh]	134,824
#3	"methods" [Subheading] OR Validation Studies as Topic [Mesh] OR Validation Study [Publication Type] OR validation[tiab] OR method*[tiab] Sort by: Publication Date	8,642,617
#2	“cohort studies” OR “cohort study” OR “cohorts design” OR “prospective cohort” OR "retrospective cohort" OR "data integration" OR bias OR ”cross study” OR ”cross studies” OR cohort studies [Mesh] OR Cross-Sectional Studies [Mesh] OR Prospective Studies[Mesh] Sort by: Publication Date	2,512,176
#1	“stratified medicine” OR biomarker* OR “precision medicine” OR “personalized medicine” OR “personalised medicine” OR “individualized Medicine“ OR “individualised Medicine“ OR “individualized therapy“ OR “individualised therapy“ OR “patient stratification” OR pharmacogenetics OR “patient specific modeling” OR “personalized clinical decision making” OR “personalised clinical decision making” OR “prediction of response” OR “prediction of responses”OR"Biomarkers"[Mesh] OR "Precision Medicine"[Mesh] Sort by: Publication Date	990,562

Table S1b. Alzheimer's Disease Searches. Embase 15/5/2020

No.	Query	Results
#16	#14 OR #15	206
#15	#13 AND [review]/lim	127
#14	#8 AND #12 AND ([cochrane review]/lim OR [systematic review]/lim OR [meta analysis]/lim)	122
#13	#8 AND #12	3250
#12	#9 OR #10 OR #11	378330
#11	dementia OR alzheimer	378330
#10	'alzheimer disease'/exp	197786
#9	'dementia'/de	116670
#8	#3 AND #6 AND #7	63646
#7	'validation study'/exp OR 'procedures'/exp OR validation:ti,ab OR method*:ti,ab	29820061
#6	#4 OR #5	1619793
#5	'cohort analysis'/exp OR 'cross-sectional study'/exp OR 'prospective study'/exp	1374626
#4	'cohort studies':ti,ab OR 'cohort study':ti,ab OR 'cohorts design':ti,ab OR 'prospective cohort':ti,ab OR 'retrospective cohort':ti,ab OR 'data integration':ti,ab OR bias:ti,ab OR 'cross study':ti,ab OR 'cross studies':ti,ab	518448
#3	#1 OR #2	543287
#2	'biological marker'/exp OR 'personalized medicine'/exp	338118
#1	'stratified medicine':ti,ab OR biomarker*:ti,ab OR 'precision medicine':ti,ab OR 'personalized medicine':ti,ab OR 'personalised medicine':ti,ab OR 'individualized medicine':ti,ab OR 'individualised medicine':ti,ab OR 'individualized therapy':ti,ab OR 'individualised therapy':ti,ab OR 'patient stratification':ti,ab OR pharmacogenetics:ti,ab OR 'patient specific modeling':ti,ab OR 'personalized clinical decision making':ti,ab OR 'personalised clinical decision making':ti,ab OR 'prediction of response':ti,ab OR 'prediction of responses':ti,ab	427868

Table S1c. Alzheimer’s Disease Searches. Web of science 15/5/2020

No.	Query	Results
# 10	#9 OR #7<br>Indexes=SCI-EXPANDED, SSCI, A&HCI, CPCI-S, CPCI-SSH, ESCI Timespan=All years	67
# 9	#8 AND #6<br>Indexes=SCI-EXPANDED, SSCI, A&HCI, CPCI-S, CPCI-SSH, ESCI Timespan=All years	40
# 8	TOPIC: (("systematic review" OR "meta analysis"))<br>Indexes=SCI-EXPANDED, SSCI, A&HCI, CPCI-S, CPCI-SSH, ESCI Timespan=All years	280,095
# 7	#5 AND #4<br>Refined by: DOCUMENT TYPES: (REVIEW)<br>Indexes=SCI-EXPANDED, SSCI, A&HCI, CPCI-S, CPCI-SSH, ESCI Timespan=All years	60
# 6	#5 AND #4<br>Indexes=SCI-EXPANDED, SSCI, A&HCI, CPCI-S, CPCI-SSH, ESCI Timespan=All years	351
# 5	TOPIC: (dementia OR alzheimer)<br>Indexes=SCI-EXPANDED, SSCI, A&HCI, CPCI-S, CPCI-SSH, ESCI Timespan=All years	265,23
# 4	#3 AND #2 AND #1<br>Indexes=SCI-EXPANDED, SSCI, A&HCI, CPCI-S, CPCI-SSH, ESCI Timespan=All years	7,402
# 3	TOPIC: ((validation OR method*))<br>Indexes=SCI-EXPANDED, SSCI, A&HCI, CPCI-S, CPCI-SSH, ESCI Timespan=All years	11,248,557
# 2	TOPIC: ((“cohort studies” OR “cohort study” OR “cohorts design” OR “prospective cohort” OR "retrospective cohort" OR "data integration" OR bias OR ”cross study” OR ”cross studies”))<br>Indexes=SCI-EXPANDED, SSCI, A&HCI, CPCI-S, CPCI-SSH, ESCI Timespan=All years	764,078
# 1	TOPIC: ((“stratified medicine” OR biomarker* OR “precision medicine” OR “personalized medicine” OR “personalised medicine” OR “individualized Medicine“ OR “individualised Medicine“ OR “individualized therapy“ OR “individualised therapy“ OR “patient stratification” OR pharmacogenetics OR “patient specific modeling” OR “personalized clinical decision making” OR “personalised clinical decision making” OR “prediction of response” OR “prediction of responses”))<br>Indexes=SCI-EXPANDED, SSCI, A&HCI, CPCI-S, CPCI-SSH, ESCI Timespan=All years	375,74

Table S1d. Alzheimer's Disease Searches. Psycinfo 15/5/2020

No.	Query	Results
S8	((TI,AB("stratified medicine" OR biomarker* OR "precision medicine" OR "personalized medicine" OR "personalised medicine" OR "individualized Medicine" OR "individualised Medicine" OR "individualized therapy" OR "individualised therapy" OR "patient stratification" OR pharmacogenetics OR "patient specific modeling" OR "personalized clinical decision making" OR "personalised clinical decision making" OR "prediction of response") AND TI,AB("cohort studies" OR "cohort study" OR "cohorts design" OR "prospective cohort" OR "retrospective cohort" OR "data integration" OR bias OR "cross study" OR "cross studies") AND TI,AB(validation OR method*)) AND TI,AB(dementia OR alzheimer)) AND TI,AB(review* OR systematic OR "meta analysis")	21
S7	TI,AB(review* OR systematic OR "meta analysis")	590,444
S6	(TI,AB("stratified medicine" OR biomarker* OR "precision medicine" OR "personalized medicine" OR "personalised medicine" OR "individualized Medicine" OR "individualised Medicine" OR "individualized therapy" OR "individualised therapy" OR "patient stratification" OR pharmacogenetics OR "patient specific modeling" OR "personalized clinical decision making" OR "personalised clinical decision making" OR "prediction of response") AND TI,AB("cohort studies" OR "cohort study" OR "cohorts design" OR "prospective cohort" OR "retrospective cohort" OR "data integration" OR bias OR "cross study" OR "cross studies") AND TI,AB(validation OR method*)) AND TI,AB(dementia OR alzheimer)	114
S5	TI,AB(dementia OR alzheimer)	97,265
S4	TI,AB("stratified medicine" OR biomarker* OR "precision medicine" OR "personalized medicine" OR "personalised medicine" OR "individualized Medicine" OR "individualised Medicine" OR "individualized therapy" OR "individualised therapy" OR "patient stratification" OR pharmacogenetics OR "patient specific modeling" OR "personalized clinical decision making" OR "personalised clinical decision making" OR "prediction of response") AND TI,AB("cohort studies" OR "cohort study" OR "cohorts design" OR "prospective cohort" OR "retrospective cohort" OR "data integration" OR bias OR "cross study" OR "cross studies") AND TI,AB(validation OR method*)	385
S3	TI,AB(validation OR method*)	863,142
S2	TI,AB("cohort studies" OR "cohort study" OR "cohorts design" OR "prospective cohort" OR "retrospective cohort" OR "data integration" OR bias OR ″cross study" OR ″cross studies")	102,458
S1	TI,AB("stratified medicine" OR biomarker* OR "precision medicine" OR "personalized medicine" OR "personalised medicine" OR "individualized Medicine" OR "individualised Medicine" OR "individualized therapy" OR "individualised therapy" OR "patient stratification" OR pharmacogenetics OR "patient specific modeling" OR "personalized clinical decision making" OR "personalised clinical decision making" OR "prediction of response")	19,287

Table S1e. Oncology Searches. Pubmed 27/3/2020

No.	Query	Results
#26	Search: #16 OR #19 Filters: English, French, German, Italian, Spanish	1,047
#22	Search: #16 OR #19 Filters: English, from 2005 - 2020	1,047
#21	Search: #16 OR #19 Filters: from 2005 - 2020	1,123
#20	Search: #16 OR #19	1,345
#19	Search: #5 AND #18	515
#18	Search: "systematic review*"	186,019
#16	Search: #1 AND #2 AND #3 AND #4 Filters: Meta-Analysis, Review	1,142
#5	Search: #1 AND #2 AND #3 AND #4	42,105
#4	Search: "Neoplasms"[Majr] OR Cancer*[tiab] OR carcinoma*[tiab] OR tumor*[tiab] OR tumour*[tiab] OR oncolo*[tiab] OR leukemia*[tiab] OR lymphoma*[tiab] OR sarcoma*[tiab]	4,008,506
#3	Search: "methods" [Subheading] OR Validation Studies as Topic [Mesh] OR Validation Study [Publication Type] OR validation[tiab] OR method*[tiab]	8,561,000
#2	Search: “cohort studies” OR “cohort study” OR “cohorts design” OR “prospective cohort” OR "retrospective cohort" OR "data integration" OR bias OR ”cross study” OR ”cross studies” OR cohort studies [Mesh] OR Cross-Sectional Studies [Mesh] OR Prospective Studies[Mesh]	2,487,093
#1	Search: “stratified medicine” OR biomarker* OR “precision medicine” OR “personalized medicine” OR “personalised medicine” OR “individualized Medicine“ OR “individualised Medicine“ OR “individualized therapy“ OR “individualised therapy“ OR “patient stratification” OR pharmacogenetics OR “patient specific modeling” OR “personalized clinical decision making” OR “personalised clinical decision making” OR “prediction of response” OR “prediction of responses”OR"Biomarkers"[Mesh] OR "Precision Medicine"[Mesh]	983,81

Table S1f. Oncology Searches. Embase 27/3/2020

No.	Query	Results
#24	#21 AND #22 AND ([english]/lim OR [french]/lim OR [german]/lim OR [italian]/lim OR [spanish]/lim)	429
#23	#21 AND #22	438
#22	[embase]/lim NOT [medline]/lim	9595932
#21	(#18 OR #19) AND [2005-2020]/py	1302
#20	#18 OR #19	1355
#19	#17 AND [review]/lim	779
#18	#4 AND #9 AND #13 AND #16 AND ([cochrane review]/lim OR [systematic review]/lim OR [meta analysis]/lim)	818
#17	#4 AND #9 AND #13 AND #16	20180
#16	#14 OR #15	5241925
#15	cancer*:ti,ab OR carcinoma*:ti,ab OR tumor*:ti,ab OR tumour*:ti,ab OR oncolo*:ti,ab OR leukemia*:ti,ab OR lymphoma*:ti,ab OR sarcoma*:ti,ab	4465029
#14	'neoplasm'/exp/mj	3703629
#13	#10 OR #11 OR #12	29597086
#12	validation:ti,ab OR method*:ti,ab	9431867
#11	'procedures'/exp	28915500
#10	'validation study'/exp	81960
#9	#5 OR #6 OR #7 OR #8	1592114
#8	'prospective study'/exp	588853
#7	'cross-sectional study'/exp	339070
#6	'cohort analysis'/exp	560295
#5	'cohort studies':ti,ab OR 'cohort study':ti,ab OR 'cohorts design':ti,ab OR 'prospective cohort':ti,ab OR 'retrospective cohort':ti,ab OR 'data integration':ti,ab OR bias:ti,ab OR 'cross study':ti,ab OR 'cross studies':ti,ab	511111
#4	#1 OR #2 OR #3	535142

#3	'personalized medicine'/exp	41477
#2	'biological marker'/exp	296253
#1	'stratified medicine':ti,ab OR biomarker*:ti,ab OR 'precision medicine':ti,ab OR 'personalized medicine':ti,ab OR 'personalised medicine':ti,ab OR 'individualized medicine':ti,ab OR 'individualised medicine':ti,ab OR 'individualized therapy':ti,ab OR 'individualised therapy':ti,ab OR 'patient stratification':ti,ab OR pharmacogenetics:ti,ab OR 'patient specific modeling':ti,ab OR 'personalized clinical decision making':ti,ab OR 'personalised clinical decision making':ti,ab OR 'prediction of response':ti,ab OR 'prediction of responses':ti,ab	420990

Table S1g. Oncology Searches. Web of Science 27/3/2020

No.	Query	Results
# 10	#9 OR #7<br>Indexes=SCI-EXPANDED, SSCI, A&HCI, CPCI-S, CPCI-SSH, ESCI Timespan=All years	590
# 9	#8 AND #6<br>Indexes=SCI-EXPANDED, SSCI, A&HCI, CPCI-S, CPCI-SSH, ESCI Timespan=All years	463
# 8	**TOPIC:** ("systematic review" OR "meta analysis")<br>Indexes=SCI-EXPANDED, SSCI, A&HCI, CPCI-S, CPCI-SSH, ESCI Timespan=All years	274,177
# 7	#4 AND #3 AND #2 AND #1<br>**Refined by: PUBLICATION YEARS:** (2020 OR 2013 OR 2006 OR 2019 OR 2012 OR 2005 OR 2018 OR 2011 OR 2017 OR 2010 OR 2016 OR 2009 OR 2015 OR 2008 OR 2014 OR 2007) AND **DOCUMENT TYPES:** (REVIEW)<br>Indexes=SCI-EXPANDED, SSCI, A&HCI, CPCI-S, CPCI-SSH, ESCI Timespan=All years	350
# 6	#4 AND #3 AND #2 AND #1<br>**Refined by: PUBLICATION YEARS:** (2020 OR 2013 OR 2006 OR 2019 OR 2012 OR 2005 OR 2018 OR 2011 OR 2017 OR 2010 OR 2016 OR 2009 OR 2015 OR 2008 OR 2014 OR 2007)<br>Indexes=SCI-EXPANDED, SSCI, A&HCI, CPCI-S, CPCI-SSH, ESCI Timespan=All years	2,34
# 5	#4 AND #3 AND #2 AND #1<br>Indexes=SCI-EXPANDED, SSCI, A&HCI, CPCI-S, CPCI-SSH, ESCI Timespan=All years	2,402
# 4	**TOPIC:** (neoplasm* OR Cancer* OR carcinoma* OR tumor* OR tumour*OR oncolo* OR leukemia* OR lymphoma* OR sarcoma*)<br>Indexes=SCI-EXPANDED, SSCI, A&HCI, CPCI-S, CPCI-SSH, ESCI Timespan=All years	3,811,935
# 3	**TOPIC:** (validation OR method*)<br>Indexes=SCI-EXPANDED, SSCI, A&HCI, CPCI-S, CPCI-SSH, ESCI Timespan=All years	11,099,593
# 2	**TOPIC:** (“cohort studies” OR “cohort study” OR “cohorts design” OR “prospective cohort” OR "retrospective cohort" OR "data integration" OR bias OR ”cross study” OR ”cross studies”)<br>Indexes=SCI-EXPANDED, SSCI, A&HCI, CPCI-S, CPCI-SSH, ESCI Timespan=All years	753,244

# 1	**TOPIC:** (“stratified medicine” OR biomarker* OR “precision medicine” OR “personalized medicine” OR “personalised medicine” OR “individualized Medicine“ OR “individualised Medicine“ OR “individualized therapy“ OR “individualised therapy“ OR “patient stratification” OR pharmacogenetics OR “patient specific modeling” OR “personalized clinical decision making” OR “personalised clinical decision making” OR “prediction of response” OR “prediction of responses”)<br>Indexes=SCI-EXPANDED, SSCI, A&HCI, CPCI-S, CPCI-SSH, ESCI Timespan=All years	368,188

Table S1h. Oncology Searches. Cochrane Library 27/3/2020

No.	Query	Results
#1	"stratified medicine":ti,ab	29
#2	biomarker*:ti,ab OR 'precision medicine':ti,ab OR 'personalized medicine':ti,ab OR 'personalised medicine':ti,ab OR 'individualized medicine':ti,ab OR 'individualised medicine':ti,ab OR 'individualized therapy':ti,ab OR 'individualised therapy':ti,ab OR 'patient stratification':ti,ab OR pharmacogenetics:ti,ab OR 'patient specific modeling':ti,ab OR 'personalized clinical decision making':ti,ab OR 'personalised clinical decision making':ti,ab OR 'prediction of response':ti,ab OR 'prediction of responses':ti,ab	34529
#3	#1 or #2	34542
#4	'cohort studies':ti,ab OR 'cohort study':ti,ab OR 'cohorts design':ti,ab OR 'prospective cohort':ti,ab OR 'retrospective cohort':ti,ab OR 'data integration':ti,ab OR bias:ti,ab OR 'cross study':ti,ab OR 'cross studies':ti,ab	125366
#5	validation:ti,ab OR method*:ti,ab validation:ti,ab OR method*:ti,ab	637228
#6	neoplasm*:ti,ab or cancer*:ti,ab OR carcinoma*:ti,ab OR tumor*:ti,ab OR tumour*:ti,ab OR oncolo*:ti,ab OR leukemia*:ti,ab OR lymphoma*:ti,ab OR sarcoma*:ti,ab	189985
#7	#3 AND #4 AND #5 AND #6 with Publication Year from 2005 to 2020, in Trials	1047
#8	REVIEW*	1621582
#9	#7 AND #8	1047
#10	"accession number" near pubmed	659110
#11	"accession number" near EMBASE	533181
#12	#10 or #11	993434
#13	#9 not #12	67

#14	clinicaltrials.gov	207571
#15	#13 NOT #14	29
#16	www.who.int	135542
#17	#15 not #16	13

Table S1i. Stroke Searches. Pubmed 3/6/2020

No.	Query	Results
#34	Search: #4 AND #28 Filters: Meta-Analysis, Review, Systematic Reviews	37
#33	Search: #4 AND #28 Filters: Review, Systematic Reviews	28
#32	Search: #4 AND #28 Filters: Systematic Reviews, from 2005 - 2020	10
#31	Search: #4 AND #28 Filters: from 2005 - 2020	1,324
#30	Search: #4 AND #28	1,412
#29	Search: #4 AND #28	1,412
#28	Search: "Cerebrovascular Disorders"[MeSH Terms] OR "ischemic attack, transient"[MeSH Terms] OR "Brain Ischemia"[MeSH Terms:noexp] OR "Brain Infarction"[MeSH Terms] OR "stroke, lacunar"[MeSH Terms] OR "Cerebral Intraventricular Hemorrhage"[MeSH Terms] OR "Cryptogenic Stroke"[Title/Abstract] OR "transient ischemic attack*"[Title/Abstract] OR "TIA"[Title/Abstract] OR "brain infarction*"[Title/Abstract] OR "Lacunar stroke"[Title/Abstract] OR "intraparenchymal hemorrhage"[Title/Abstract] OR "intraventricular hemorrhage"[Title/Abstract] OR "ischemic stroke"[Title/Abstract] OR TIA[Title/Abstract] OR ischemic[Title/Abstract] OR ischaemic[Title/Abstract] OR stroke*[Title/Abstract] OR "CEREBROVASCULAR ACCIDENT*"[Title/Abstract] OR cva[Title/Abstract]	631,303
#4	Search: #1 AND #2 AND #3	33,207
#3	Search: "methods" [Subheading] OR Validation Studies as Topic [Mesh] OR Validation Study [Publication Type] OR validation[tiab] OR method*[ti]	4,256,076
#2	Search: “cohort studies” OR “cohort study” OR “cohorts design” OR “prospective cohort” OR "retrospective cohort" OR "data integration" OR bias OR ”cross study” OR ”cross studies” OR cohort studies [Mesh] OR Cross-Sectional Studies [Mesh] OR Prospective Studies[Mesh]	2,521,666
#1	Search: “stratified medicine” OR biomarker* OR “precision medicine” OR “personalized medicine” OR “personalised medicine” OR “individualized Medicine“ OR	995,072

	“individualised Medicine“ OR “individualized therapy“ OR “individualised therapy“ OR “patient stratification” OR pharmacogenetics OR “patient specific modeling” OR “personalized clinical decision making” OR “personalised clinical decision making” OR “prediction of response” OR “prediction of responses”OR"Biomarkers"[Mesh] OR "Precision Medicine"[Mesh]	

Table S1j. Stroke Searches. Embase 3/6/2020

No.	Query	Results
#22	(#18 OR #19) AND [humans]/lim AND [2005-2020]/py	168
#21	(#18 OR #19) AND [humans]/lim	171
#20	#18 OR #19	177
#19	#17 AND [review]/lim	101
#18	#8 AND #16 AND ([cochrane review]/lim OR [systematic review]/lim OR [meta analysis]/lim)	104
#17	#8 AND #16	3,037
#16	#9 OR #10 OR #11 OR #12 OR #13 OR #14 OR #15	780,035
#15	'brain ventricle hemorrhage'/exp	1,027
#14	'lacunar stroke'/exp	3,336
#13	'brain infarction'/exp	74,495
#12	'brain ischemia'/exp	185,994
#11	'transient ischemic attack'/exp	38,968
#10	'cerebrovascular disease'/de	63,275
#9	'cryptogenic stroke':ti,ab OR 'transient ischemic attack*':ti,ab OR 'tia':ti,ab OR 'brain infarction*':ti,ab OR 'lacunar stroke':ti,ab OR 'intraparenchymal hemorrhage':ti,ab OR 'intraventricular hemorrhage':ti,ab OR 'ischemic stroke':ti,ab OR tia:ti,ab OR ischemic:ti,ab OR ischaemic:ti,ab OR stroke*:ti,ab OR 'cerebrovascular accident*':ti,ab OR cva:ti,ab	650,270
#8	#3 AND #6 AND #7	63,599
#7	'validation study'/exp OR 'procedures'/exp OR validation:ti,ab OR method*:ti	29,333,023

#6	#4 OR #5	1,636,917
#5	'cohort analysis'/exp OR 'cross-sectional study'/exp OR 'prospective study'/exp	1,390,377
#4	'cohort studies':ti,ab OR 'cohort study':ti,ab OR 'cohorts design':ti,ab OR 'prospective cohort':ti,ab OR 'retrospective cohort':ti,ab OR 'data integration':ti,ab OR bias:ti,ab OR 'cross study':ti,ab OR 'cross studies':ti,ab	523,306
#3	#1 OR #2	547,946
#2	'biological marker'/exp OR 'personalized medicine'/exp	341,290
#1	'stratified medicine':ti,ab OR biomarker*:ti,ab OR 'precision medicine':ti,ab OR 'personalized medicine':ti,ab OR 'personalised medicine':ti,ab OR 'individualized medicine':ti,ab OR 'individualised medicine':ti,ab OR 'individualized therapy':ti,ab OR 'individualised therapy':ti,ab OR 'patient stratification':ti,ab OR pharmacogenetics:ti,ab OR 'patient specific modeling':ti,ab OR 'personalized clinical decision making':ti,ab OR 'personalised clinical decision making':ti,ab OR 'prediction of response':ti,ab OR 'prediction of responses':ti,ab	431,979

Table S1k. Stroke Searches. Web of Science 3/6/2020

No.	Query	Results
# 11	#10 OR #8<br><br>Indexes=SCI-EXPANDED, SSCI, A&HCI, CPCI-S, CPCI-SSH, ESCI Timespan=All years	61
# 10	#9 AND #7<br><br>Indexes=SCI-EXPANDED, SSCI, A&HCI, CPCI-S, CPCI-SSH, ESCI Timespan=All years	51
# 9	TOPIC: ((("systematic review" OR "meta analysis")))<br><br>Indexes=SCI-EXPANDED, SSCI, A&HCI, CPCI-S, CPCI-SSH, ESCI Timespan=All years	282,750
# 8	#5 AND #4<br>Refined by: PUBLICATION YEARS: (2020 OR 2016 OR 2012 OR 2008 OR 2019 OR 2015 OR 2011 OR 2007 OR 2018 OR 2014 OR 2010 OR 2006 OR 2017 OR 2013 OR 2009) AND DOCUMENT TYPES: (REVIEW)<br>Indexes=SCI-EXPANDED, SSCI, A&HCI, CPCI-S, CPCI-SSH, ESCI Timespan=All years	33
# 7	#5 AND #4	395

	Refined by: PUBLICATION YEARS: (2020 OR 2016 OR 2012 OR 2008 OR 2019 OR 2015 OR 2011 OR 2007 OR 2018 OR 2014 OR 2010 OR 2006 OR 2017 OR 2013 OR 2009)<br>Indexes=SCI-EXPANDED, SSCI, A&HCI, CPCI-S, CPCI-SSH, ESCI Timespan=All years	
# 6	#5 AND #4<br><br>Indexes=SCI-EXPANDED, SSCI, A&HCI, CPCI-S, CPCI-SSH, ESCI Timespan=All years	396
# 5	TOPIC: ("Cryptogenic Stroke" OR "transient ischemic attack*" OR "TIA" OR "brain infarction*" OR "Lacunar stroke" OR "intraparenchymal hemorrhage" OR "intraventricular hemorrhage" OR "ischemic stroke" OR TIA OR ischemic OR ischaemic OR stroke* OR “CEREBROVASCULAR ACCIDENT*” OR cva)<br><br>Indexes=SCI-EXPANDED, SSCI, A&HCI, CPCI-S, CPCI-SSH, ESCI Timespan=All years	515,470
# 4	#3 AND #2 AND #1<br><br>Indexes=SCI-EXPANDED, SSCI, A&HCI, CPCI-S, CPCI-SSH, ESCI Timespan=All years	7,499
# 3	TOPIC: (((validation OR method*)))<br><br>Indexes=SCI-EXPANDED, SSCI, A&HCI, CPCI-S, CPCI-SSH, ESCI Timespan=All years	11,303,677
# 2	TOPIC: (((“cohort studies” OR “cohort study” OR “cohorts design” OR “prospective cohort” OR "retrospective cohort" OR "data integration" OR bias OR ”cross study” OR ”cross studies”)))<br><br>Indexes=SCI-EXPANDED, SSCI, A&HCI, CPCI-S, CPCI-SSH, ESCI Timespan=All years	768,507
# 1	TOPIC: (((“stratified medicine” OR biomarker* OR “precision medicine” OR “personalized medicine” OR “personalised medicine” OR “individualized Medicine“ OR “individualised Medicine“ OR “individualized therapy“ OR “individualised therapy“ OR “patient stratification” OR pharmacogenetics OR “patient specific modeling” OR “personalized clinical decision making” OR “personalised clinical decision making” OR “prediction of response” OR “prediction of responses”)))<br><br>Indexes=SCI-EXPANDED, SSCI, A&HCI, CPCI-S, CPCI-SSH, ESCI Timespan=All years	378,951

Table S1l. **Grey literature search**

Date of search	Source	Url	Relevant document identified (Y/N)	SEARCH STRATEGY
May 11, 2020	Heads of Medicines Agencies	https://www.hma.eu	Y	
May 11, 2020	EUROPEAN ALLIANCE FOR PERSONALIZED MEDICINE	https://www.euapm.eu	N	
May, 15	European comission	https://ec.europa.eu/	Y	
March 11, 2020	FDA	https://www.fda.gov/	Y	
June 4, 2020	Explore the British Library	http://explore.bl.uk	Y	"Personalised medicine"
June 4, 2020	ESMO	https://www.esmo.org/	N	("personalised medicine"+"legal"); ("personalised medicine" + "regulation") filtered by "ethics, legal..."
June 5, 2020	OpenGrey	http://www.opengrey.eu/	Y	"Personalised medicine"; "cohorts validation"; "cohorts stratification" ("legal" + "personalised medicine"); ("ethics" + "personalised medicine"); ("data management"+"personalised medicine")
June 5, 2020	Healthcare Management Information Consortium (HMIC)	https://www.ovid.com/	N	

June 5, 2020	National Technical Information Service (NTIS)	www.ntis.gov/		
June 5, 2020	European Medicines Agency (EMA)	https://www.ema.europa.eu/	Y	Personalised medicine; ("legal frame" + "personalised medicine"); ("ethics" + "personalised medicine")
June 5, 2020	Canadian Agency for Drugs and Technologies in Health (CADTH).	https://www.cadth.ca/	N	personalised medicine
June 5, 2020	WHO (europe)	http://www.euro.who.int/en/what-we-do/data-and-evidence/health-evidence-network-hen/publications/by-keyword	N	personalised medicine
June 5, 2020	National Institute for health and Care Excelence	https://www.nice.org.uk/	N	

Table S2. Summary of included reviews from PubMed, EMBASE, Web of Science, Psycinfo, and the Cochrane Library.

**Author(s)**	**Disease analysed**	**Principal outcome related to the aim of this scoping review**	**Q1**	**Q2**	**Q3**	**Q4**	**Q5**	**Q6**	**Q7**	**Q8**
Ard 2011[32]	Alzheimer's disease	Summary of methods to estimate the appropriate sample size.		x	x					
Habes 2020 [47]	Alzheimer's disease	Summary of clusters generated from different kind of dementias, including clustering methods and their challenges in Alzheimer.			x			x		
Baker 2006 [23]	Cancer	An analysis of different kind of studies: preliminary performance, retrospective performance, prospective performance and cancer screening evaluation; methodological issues and the strengths and limitations of these studies.	x	x	x					
Hackl 2010 [33]	Cancer	A review about computational approaches for data integration: data aggregation; integrative data analysis, including methodological aspects; biomolecular pathways and network reconstruction; and mathematical modelling.		x	x	x				
Zalcman 2011 [25]	Cancer (non-small cell lung cancer)	Summary of the major methodological issues concerning prognostic biomarkers in non-small cell lung cancers (NSCLCs), focused on retrospective studies and on the design of validation studies.	x						x	
Rundle 2012 [64]	Cancer	An analysis of case–control study designs in relation to biomarker discovery studies.							x	
Hayes 2013 [30]	Cancer	Collection of strategies to generate high levels of evidence of clinical utility; report of guidelines for tumour marker studies; description of regulatory oversight of tumour biomarker tests and some case studies; limitations of prospective approaches.	x							
Pesch, 2014 [29]	Cancer	Description of the principles of biomarker research in prospective studies; examples of diagnostic tumour markers and their source of bias.	x							x
Verma 2017 [36]	Cancer	Characterization of the microbiome in different tumour types to identify biomarkers of risk, progression, and prognosis; mechanistic and technical challenges in the field of microbiome and cancer epidemiology.			x					
Abraham 2016 [35]	Cancer	Summary of the limitations in hepatocellular carcinoma staging and proposal of solution using personalised medicine.			x			x	x	
Borad 2017 [28]	Cancer	Description of the general design of studies and trials in cancer.	x		x					
Hurgobin 2018 [58]	Cancer (lung cancer)	Summary of basic concepts in bioinformatics and genomic data analysis, including a description of tools and approaches to conduct data integration.				x				

Karczewski 2018 [38]	Cancer	A discussion on the potential of combining diverse types of data and the utility of this approach in human health and disease; technical challenges to clinical implementation of integrative omics.			x	x				
SHahinas 2018 [26]	Cancer (squamous oral cell carcinoma)	Analysis of the methods used in molecular marker prognosis studies of oral squamous cell carcinoma and of the risk of bias. The analysis is based in Quality in Prognosis Studies tool.	x							x
Bradley 2019 [27]	Cancer	Description of methods used in predictors of survival in cancer patients.	x						x	x
Zanfardino 2019 [37]	Cancer	Examination of the role of radiogenomics in cancer phenotype definition; current state of radiogenomics data integration in public repository; summary of the challenges and limitations of including radiomics in MultiAssayExperiment MAE.			x	x				
Parikh 2019 [31]	Cancer	Characteristics and limitations of retrospective studies; regulation of predictive analytics in medicine.	x							
López de Maturana 2019 [59]	Cancer	Summary of integration methods of omics and non-omics data, and the study of their challenges.				x				
Hamada 2019 [24]	Cancer	A discussion and analysis of data integration in the fields of microbiology, molecular pathology and epidemiology.	x		x	x				X
Cazaly 2019 [39]	Cancer	An overview of recent data analysis approaches to integrate various omics layers in order to understand epigenetic mechanisms of complex diseases, such as obesity and cancer.			x	x				
Lin 2020 [34]	Cancer	Methodologies and applications for multidimensional data integration and computational modelling; discussion about the future perspectives and challenges for prostate cancer systems medicine and holistic healthcare.			x	x				
Moons 2009 [21]	Multiple Diseases	A discussion about challenges and lack of multivariable prognostic models.	x	x	x					
Ransohoff 2010 [42]	Multiple Diseases	Summary of main sources of bias in precision medicine.			x					X
Flynn 2011 [20]	Multiple Diseases	Description of different stratification and validation designs and their challenges.	x		x				x	
Ioannidis 2011 [40]	Multiple Diseases	An analysis of the issues that are specific to proteomic biomarkers.			x				x	
Desai 2011 [54]	Multiple Diseases	Guidelines to deal with missing data (multiple imputation...)				x				

Austin 2011 [63]	Multiple Diseases	Description of four different propensity score methods: matching on the propensity score; stratification on the propensity score; inverse probability of treatment weighting using the propensity score; and covariate adjustment using the propensity score.						x	x	
Sung 2012 [18]	Multiple Diseases	An analysis of different strategies in the different stages of biomarkers discovery.	x		x	x				X
Bouwmeester [22]	Multiple Diseases	A review of articles focused on clinical prediction research, based on a comprehensive item list to score, conduct and report the studies.	x	x	x	x			x	
Anderson [57]	Multiple Diseases	An analysis of different analytical methods used for biomarker discovery and validation; discussion on the utility of single biomarkers due to the existence of biomarker panels and the role of large-scale biomarker consortia; analysis of risk of bias and their sources.				x			x	X
Ensor 2014 [19]	Multiple Diseases	Description of the limitations of retrospective cross-sectional data in case-control approaches. Discussion on the main areas of concern in the biomarker validation process: correlated observation multiplicity, multiple clinical endpoints and selection bias.	x							X
Schrodi 2014 [46]	Multiple Diseases	Summary of the current state of disease susceptibility mapping and the pharmacogenetics efforts for risk prediction; description of the methods used to construct and evaluate genetic-based predictive models and discussion of their applications.			x	x				
Gligorijevic 2015 [53]	Multiple Diseases	Summary of integrative methods for disease subtyping, biomarkers discovery, and drug repurposing; list of the tools available. Given the ever-growing nature of these big data, key issues that big data integration methods will face is highlighted.				x				
Ritchie [41]	Multiple Diseases	Summary of approaches for data integration, including meta-dimensional and multi-staged analyses.			x	x				
López de Maturana 2016 [53]	Multiple Diseases	Description of the omics and non-omics data integration from an epidemiological point of view by considering the “massive” inclusion of variables in risk assessment and predictive models; proposal of analytical strategies that allow considering both omics and non-omics data in the models; a review about challenges embedding this type of research.				x				
Bersanelli 2016 [56]	Multiple Diseases	A review of the most advanced strategies for integrating multi-omics datasets, focusing on mathematical and methodological aspects.				x				
Casamassimi 2017 [45]	Multiple Diseases	A review about novel insights about integration and analysis methods, focusing on the integration of transcriptomics with other -omics.			x	x				

Ioannidis 2017 [65]	Multiple Diseases	An analysis of the elements in the current biomarker pipeline and the pipeline as a whole, focused on the main reasons for the failure of biomarkers and how these failures can be overcome.							x	
Grapov 2018 [44]	Multiple Diseases	Summary of analysis and –omics integration methods based on deep learning.			x	x				
Parimbelli 2018 [55]	Multiple Diseases	Summary of methods for the stratification of cohorts and their analysis, and the current use of them.				x		x		
Mirza 2019 [52]	Multiple Diseases	Summary of machine learning (ML) approaches that can address important challenges in personalised medicine.				x				
Angione 2019 [43]	Multiple Diseases	Description of model-building and data integration methods that are being used to generate patient-specific models as well as state-of-the-art machine learning methods.			x	x				
Subramanian 2019 [50]	Multiple Diseases	Summary of tools and methods to adopt integrative approaches in order to analyse multiple omics data and to summarize their ability to address applications such as disease subtyping or biomarker prediction.				x				
Swerdlow 2015 [48]	Stroke (Cardiovascular diseases)	A review of the recent advances in the contribution of genetics to cardiovascular disease (CVD). The review includes information about pharmacogenetics, personalised genetic testing at the point of care, large-scale sequencing projects and emerging -omics technologies for the knowledge of the genetic basis of CVD.			x	x				
Edwardson 2020 [49]	Stroke	Summary of pros and cons of two techniques to match control-patient; statistical tools to gauge balance between patients and controls (in validation cohorts); techniques used for the recruitment of controls.			x				x	

Q1 Prospective and retrospective cohorts: pros and cons.

Q2 Definition of the optimal size of stratification/validation cohorts and prerequisites and methods used for the integration of multiple retrospective cohorts.

Q3 Type of data generated.

Q4 Tools used for data management and multimodal data analysis in personalised medicine, and their gaps.

Q5 Quality of data of cohorts needed to obtain a biomarker or multimodal data profiling and the requirements to monitor the collection of associated clinical data.

Q6 Current designs for the stratification (or clustering).

Q7 Methods and tools used in validation cohort building and their gaps.

Q8 Methods for the evaluation of the risk of bias.

Table S3. Summary of included articles from grey literature.

<table>
<tr><th>Number of question</th><th>Title</th><th>Provider institution</th><th>Information included in the document</th></tr>
<tr><td rowspan="5">Q9. Outlook of data generation seen as (CE-labelled) in-vitro diagnostics.</td><td>In vitro diagnostic medical devices. Directive 98/79/EC</td><td>European commission 2020 [70]</td><td rowspan="2">These documents explain the new regulation that will be applied in the European Union before May 2022.</td></tr>
<tr><td>TUVSUD. In vitro diagnostic medical device regulation (IVDR)</td><td>TÜV SÜD 2017 [69]</td></tr>
<tr><td>Diagnostics in vitro.</td><td>Food and Drug Administration 2019 [66]</td><td>This document defines In-Vitro diagnostics and explains their relevance in precision medicine.</td></tr>
<tr><td>Companion Diagnostics.</td><td>Food and Drug Administration 2018 [68]</td><td rowspan="2">These documents explain the type of new drugs accepted by FDA, highlighting the importance of the ones in personalised medicine.</td></tr>
<tr><td>Principles for Codevelopment of an In Vitro Companion Diagnostic Device with a Therapeutic Product.</td><td>Food and Drug Administration 2018 [67]</td></tr>
</table>

Table S4. Pros and cons of prospective and retrospective study designs.

	Prospective		Retrospective	
	Pros	Cons	Pros	Cons
Oncology	Better definition of included population that developed tumours (diminution of selection bias).	Bias due to overdiagnosis (If the endpoint is cancer on biopsy).	Quick and valid way.	Propensity to recall and selection biases (in single-cohort studies lacking a control arm).
	Recall bias is reduced.	Large sample size requirement.	No requirement for follow-up of participants.	Possible reinforcement of existing biases in clinical care.
	Preferred design for prognosis studies.	Lead-time bias. A marker detects a disease earlier than usual care but does not result in a lower disease-specific mortality.	Lower costs.	Need to be prospectively validated.
		Length bias. Slowly growing tumours may be captured by diagnostic markers better than rapidly growing cancers, which more frequently appear between screening intervals.	Ability to investigate rare tumours.	Compromised generalizability of the predictive model (Due to lack of data from unrepresented populations).
		Healthy-volunteer and spectrum bias. All human studies are voluntary where subjects with a healthy lifestyle, like non-smokers, are more likely to take part in health research. Spectrum bias is observed when subjects are healthier than the target	Enables optimal measurement of predictors and outcome.	Prognostic estimates cannot be revised.

		population in which the marker will be later applied.		
			Longer follow-up time easily available.	Normally, poorer quality data.
				Lower grade of security in assignment of causality
				Heterogeneity of histologic tumour types.
				Inflation of value of broad-based NGS (Next-generation sequencing) profiling in the setting of inclusion of patients with well characterized alterations in reported studies.
Multiple diseases	High control of all aspects of the experiment.	Expensive	Wealth of data that exist.	The assignment of causality is difficult.
	Regulatory preference for prospectively designed studies.	A lot of time required.	Longer follow-up period.	Normally, poorer quality data.
	Optimal measurement of predictors and outcome.			

Table S5. Definition and pros and cons of specific prospective designs found in oncology reviews.

**Study design**	**Sampling of specimens**	**Auxiliary data**	**Cons**	**Pros**
Population-based cohort in the general population.	Sampling at baseline for a variety of purposes. Repeated sampling is frequently not possible, e.g. due to budgetary constraints. Biobanking has become an integral part of these studies.	·A large set of data about the subject and the sample. ·Laboratory parameters not specifically selected for marker research.	·Not conducted in a pre-defined at-risk population. ·Not aimed at marker research. ·No serial investigations. ·Insufficient cases with pre-diagnostic samples collected within 12 months prior to diagnosis.	·Well-conducted according to good epidemiological practice (GEP). ·Supervision of the recruitment of subjects and samples. ·Providing reference samples for case-control comparisons.
Screening cohort in the target population at risk to detect the chance outcome.	Serial collection of pre-diagnostic samples. Biobanking, not an integral part of screening in clinical settings.	·Limited information about the subject and sample. ·Laboratory parameters frequently not according to common SOPs.	·Efforts needed to implement GEP into practice. ·Lack of comparison of the marker(s) with the usual care. ·Voluntary screens with limited compliance to attend the screens regularly.	·Serial screens in the target population. ·Suitable for add-on studies on new markers. ·Well-described case group if the diagnostic workup is performed in the center that conducts the screening.
Randomized controlled trial.	Repeated sampling. Biobanking is not an integral part in clinical settings.	·Depending on study protocol.	·Long-lasting and very costly. ·Markers in clinical practice contaminate usual care arm.	·Gold standard in marker research with the highest standards for conducting and reporting.

“Prospective, retrospective” studies.	Use of archived specimens collected from patients who have participated in other prospective studies.	·Proposed solution to cons:<br><br>Recently, an international registry has been established so that investigators can prospectively document their intention to perform a tumor biomarker study, in a similar manner to “clinicaltrials.gov”. Thus, regardless of which strategy is pursued, an investigator can document that the study protocol, methods and analytical techniques were prospectively considered.	·Specimen availability (it is almost always far less than 100%).<br>·Pre-analytical concerns and assay failure.<br>·Under-powering, since the parent trial is almost always powered for the main therapeutic effect, not the tumor biomarker sub-analysis.<br>·They need to be prospectively validated.	

Table S6. Methods for validation defined in the oncology reviews found.

External validation.<br>Data used in external validation are different from the data used to generate the results.
Internal validation.<br>The analysis is conducted using the same data that were used to generate the results.
Selection of a set of patients from the whole sample.<br>A training set of patients, randomly selected from the whole series of patients, allows us to obtain a multivariable prediction model, including the biomarkers of interest and the clinical predictors. Later, this model is tested without any change in the rest of the original series of patients, used as a validation set. Such a strategy needs a large initial sample size to get precise estimates.
'Leave-one-out' cross-validation approach.
Bootstrap method.<br>Bootstrapping consists of drawing samples (with replacement) from the original data set to generate a large number of training sets (several hundred) of the same size as the original sample. A prediction model is developed on each training set and tested on the original data. Validation results are the average of all training sets.
Interactive Bayesian model and artificial neural networks (ANN).<br>Both methods use random split technique between training and validation data sets.
